# The Alternation of Gray Matter Morphological Topology in Drug-Naïve Tourette’s Syndrome in Children

**DOI:** 10.3389/fnagi.2022.873148

**Published:** 2022-05-27

**Authors:** Yi Liao, Xiuli Li, Fenglin Jia, Yuexin Jiang, Gang Ning, Xuesheng Li, Chuan Fu, Hui Zhou, Xuejia He, Xiaotang Cai, Haibo Qu

**Affiliations:** ^1^Department of Radiology, West China Second University Hospital, Sichuan University, Chengdu, China; ^2^Key Laboratory of Birth Defects and Related Diseases of Women and Children (Sichuan University), Ministry of Education, Chengdu, China; ^3^Department of Radiology, West China Hospital, Sichuan University, Chengdu, China; ^4^Department of Radiology, Chengdu Office Hospital of People’s Government of Tibet Autonomous Region, Chengdu, China; ^5^Department of Rehabilitation, West China Second University Hospital, Sichuan University, Chengdu, China

**Keywords:** magnetic resonance imaging, morphological topology, Tourette’s syndrome, children, gray matter

## Abstract

Tourette syndrome (TS) is a neurodevelopment disorder characterized by motor and phonic tics. We investigated the topological alterations in pediatric TS using morphological topological analysis of brain structures. We obtained three-dimensional T1-weighted magnetic resonance imaging (MRI) sequences from 59 drug-naïve pediatric patients with TS and 87 healthy controls. We identified morphological topographical alterations in the brains of patients with TS compared to those of the healthy controls *via* GRETNA software. At the global level, patients with TS exhibited increased global efficiency (E_*glob*_) (*p* = 0.012) and decreased normalized characteristic path length (λ) (*p* = 0.027), and characteristic path length (Lp) (*p* = 0.025) compared to healthy controls. At the nodal level, we detected significant changes in the nodal betweenness, nodal degree, and nodal efficiency in the cerebral cortex-striatum-thalamus-cortex circuit. These changes mainly involved the bilateral caudate nucleus, left thalamus, and gyri related to tics. Nodal betweenness, nodal degree, and nodal efficiency in the right superior parietal gyrus were negatively correlated with the motor tic scores of the Yale Global Tic Severity Scale (YGTSS) (*r* = −0.328, *p* = 0.011; *r* = −0.310, *p* = 0.017; and *r* = −0.291, and *p* = 0.025, respectively). In contrast, nodal betweenness, nodal degree, and nodal efficiency in the right posterior cingulate gyrus were positively correlated with the YGTSS phonic tic scores (*r* = 0.353, *p* = 0.006; *r* = 0.300, *p* = 0.021; *r* = 0.290, and *p* = 0.026, respectively). Nodal betweenness in the right supplementary motor area was positively correlated with the YGTSS phonic tic scores (*r* = 0.348, *p* = 0.007). The nodal degree in the right supplementary motor area was positively correlated with the YGTSS phonic tic scores (*r* = 0.259, *p* = 0.048). Diagnosis by age interactions did not display a significant effect on brain network properties at either the global or nodal level. Overall, our findings showed alterations in the gray matter morphological networks in drug-naïve children with TS. These findings enhance our understanding of the structural topology of the brain in patients with TS and provide useful clues for exploring imaging biomarkers of TS.

## Introduction

Tourette syndrome (TS) is a neurodevelopmental disorder characterized by multiple motor tics and at least one vocal tic present for greater than 1 year (American Psychiatric Association [APA], 2013). The symptoms wax and wane in frequency (American Psychiatric Association [APA], 2013). The age of onset is before 18 years (American Psychiatric Association [APA], 2013). Motor tics start at the age of 3–8 years, while phonic tics can begin as early as 3 years, but typically they follow the onset of motor tics by several years (Leckman and Cohen, 1999; Leckman, 2002). Some symptoms gradually decrease with age, whereas others persist into adulthood. TS may be comorbid with autism spectrum disorder (ASD), obsessive-compulsive disorder (OCD), attention deficit hyperactivity disorder (ADHD), or other mental disorders. The symptoms of TS are complex, recurrent, difficult to treat, and are often accompanied by a variety of behavioral disorders, which greatly impact children’s ability to learn, live, and interact socially (Robertson, 2000). The neural mechanisms, by which tics arise, are not fully understood. The pathogenesis of TS remains unclear, and genetic defects can lead to neuroanatomical abnormalities and neurobiochemical dysfunction. Most scholars speculate that the disease is related to neuronal dysfunction in the basal ganglia, prefrontal lobe, and limbic system, and its onset is related to various factors, such as psychiatric, environmental, genetic, and epigenetic factors, as well as embryonic development and infections, biochemical metabolism, and improper medication. Dysregulation of the actions of brain dopamine and 5-hydroxytryptamine and their interactions are currently considered to be related to tics, and abnormalities in the cerebral cortex-striatum-thalamus-cortex (CSTC) circuit are associated with the development of TS (Leckman, 2002).

In recent years, MRI has rapidly developed. It is non-invasive and has a high temporal and spatial resolution. MRI can provide physiopathological information at multiple levels, from macroscopic tissue morphology to microscopic subcellular structures, from blood flow and energy metabolism to functional changes in brain regions for the diagnosis, prediction, treatment, and evaluation of diseases. The development of multimodal neuroimaging with MRI has provided an opportunity to achieve a breakthrough in elucidating the neurological mechanisms of neuropsychiatric disorders and to explore objective indicators for clinical diagnosis and treatment (Gong, 2020). This provides strong technical support for investigating the structure and function of the brain. Several studies have explored structural brain imaging changes in patients with TS compared to those of healthy controls. Kong et al. (2020) conducted a surface-based study and detected alterations in cortical thickness, sulcus, cortical curvature, and local gyrification index in 52 patients with TS compared with those structures in 52 healthy controls. Greene et al. conducted a study on altered gray matter and white matter in 103 children with TS and 103 healthy controls. They observed an increase in the volume of the gray matter of the posterior thalamus, hypothalamus, and midbrain and a decrease in the volume of the white matter bilaterally in the orbital and medial prefrontal cortex using the voxel-based morphometry approach (Greene et al., 2017). Draper et al. (2016) reported that premonitory sensory phenomena are negatively related to the thicknesses of the insula and sensorimotor cortex. White matter abnormalities of the cortical-striatal-pallido-thalamic were explored in a study by Worbe et al. (2015) and they determined that there was an increase in the brain volume in the thalamus and right cingulum bundle. A decrease in white matter volume in the right frontal pole was reported by Liu et al. (2013), and brain volume changes were observed in the bilateral frontal and left temporal lobes. A reduction in gray matter volume in the bilateral frontal lobe and cingulum was reported by Draganski et al. (2010). Müller-Vahl et al. (2009) detected both gray matter volume and white matter volume reductions in the frontal lobe. Increases were found in the left middle frontal gyrus and left sensorimotor areas. The YGTSS scores were negatively correlated with the gray matter volume.

The aforementioned studies demonstrated gray and white matter alterations in brain regions involved CSTC in pediatric patients with TS. However, there have been no structure-based topological analyses on TS in children. A novel approach called structural covariance network (SCN) analysis has been utilized for brain structure analysis in 3D T1-weighted imaging (Seidlitz et al., 2018; Sun et al., 2018). The analysis was performed using structural brain data to construct a structural network with correlations (He et al., 2007; Alexander-Bloch et al., 2013). The analysis was performed using structural brain data to construct correlations between structural networks. It is a useful tool for morphological topological analysis of the brain (Tijms et al., 2012). We aimed to explore the morphological topological alterations in the gray matter of pediatric patients with TS. This study hypothesized that gray matter morphological topology differed in pediatric patients with TS from healthy controls.

## Materials and Methods

### Participants

Participants were recruited from July 2015 to June 2020 at West China Second University Hospital, Sichuan University. This study was approved by the Ethics Committee of the West China Second University of Sichuan University. The legal guardians of all pediatric patients provided written informed consent. A total of 59 pediatric and adolescent patients with TS were recruited from West China Second University Hospital, Sichuan University. The diagnosis criteria of pediatrics were made according to the Diagnostic and Statistical Manual of Mental Disorders-5 (*DSM-V*) (American Psychiatric Association [APA], 2013). The *DSM-V* criteria for Tourette disorder are as follows (American Psychiatric Association [APA], 2013): (i) both multiple motor and one or more vocal tics were present at some time during the illness, though not necessarily concurrently (a tic is a sudden, rapid, recurrent, non-rhythmic, stereotyped motor movement, or vocalization); (ii) the tics may wax and wane in frequency, but have persisted for more than 1 year since the onset of the first tic; (iii) onset occurred before 18 years of age; and (iv) the disturbance is not due to the direct physiological effects of a substance (e.g., cocaine) or a general medical condition (e.g., Huntington’s disease or post-viral encephalitis). The exclusion criteria were as follows: (1) concomitant with other neurological, psychiatric, or metabolic diseases; (2) MRI scans indicated structural or signal abnormalities of intracranial lesions; (3) claustrophobia or otherwise unsuitable for MRI scans; and (4) medication used for TS treatment. Initially, 85 patients with TS were recruited. Fourteen patients with TS were concomitant with ADHD. Three patients with TS were concomitant with OCD. No patient with TS were concomitant with OCD and ADHD or ASD. One of the patients with TS had a combination of abnormal thyroid function. Six patients were excluded because of image quality issues. Two patients fail to persist through the examination. All above 26 patients were excluded. Finally, 59 patients with TS were included in this study.

A total of 87 age- and gender-matched healthy pediatric controls participated in this study. The exclusion criteria were as follows: (1) neurological, psychiatric, or other metabolic diseases; (2) MRI scans indicated structural or signal abnormalities of intracranial lesions; (3) claustrophobia or otherwise unsuitable for MRI scans; and (4) a history of mental disorders.

### Data Acquisition

Clinical data are collected using a standardized process for assessment and collection by clinicians. All the participants are drug- naïve.

Imaging data were collected from pediatric patients with TS and healthy controls (HCs). All participants were scanned using 1.5T MRI and 3D T1 structural images were obtained. The scan parameters for T1-weighted imaging were as follows: echo time = 4.6 ms; repetition time = 9.6 ms; flip angle = 8°; slice thickness = 1 mm; no interslice gap; voxel size = 0.8 × 0.8 × 0.6 mm^3^; field of view = 240 mm × 240 mm; and matrix size = 256 × 256. The total number of slices was 162.

### Data Preprocessing

Magnetic resonance images were evaluated by two expert radiologists (YL and HQ with 11 years and 16 years of MRI diagnosis experience, respectively) for artifacts, structural, or signal abnormalities, and 6 subjects were excluded from the study. Routine image processing, such as spatial smoothing and spatial normalization, was performed using the Statistical Parametric Mapping (SPM) software package (version 12^1^) for voxel-based morphometry (VBM) analysis (Ashburner et al., 2000). Specifically, tissue segmentation was performed with structural images (Ashburner and Friston, 2005) and the segmented gray matter (GM) of each subject was non-linearly co-registered to the generated study-specific template using Diffeomorphic Anatomical Registration Through Exponentiated Lie Algebra (DARTEL) software (Ashburner, 2007). Co-registered images were then transformed to the standard Montreal Neurological Institute (MNI) space, the same space as the brain parcellation, modulated by the Jacobian determinants derived from the spatial normalization to preserve tissue volume after warping, resampled to a 2-mm isotropic resolution, and finally smoothed using a Gaussian kernel with a 6-mm full-width half-maximum (FWHM).

### Construction of Gray Matter Morphological Networks

For each subject, the nodes of the GM network were defined as the 90 cortical regions of interest (ROIs) (Tzourio-Mazoyer et al., 2002) utilizing the automated anatomical labeling (AAL) algorithm. The edges were defined as the Kullback–Leibler divergence-based similarity (KLS) measurement (Kong et al., 2014) of morphological distributions between different cortical regions, which was previously described in detail (Kong et al., 2014, 2015; Wang et al., 2016). Briefly, kernel density estimation (KDE) (Wang et al., 2016) was used to estimate the probability density functions (PDFs) of image intensities within each cortical region from the AAL method, with the KDE bandwidths estimated by Scott’s rule (Scott, 2015) using public Matlab codes (function: kde^2^). The similarity of image intensity PDFs of different cortical regions was quantified as the Kullback–Leibler (KL) divergence, which is a probability theory index calculating the differences between two probability distributions (Burnhan and Anderson, 2002). KLS values range between 0 and 1, where 1 represents an identical distribution for the two regions. The KL-based similarity values between all possible pairs of 90 cortical regions formed a 90×90 similarity matrix, in which each element represents the similarity of morphological distributions between two cortical regions.

The GM structural network properties were calculated using the Matlab-based GRETNA software (Wang et al., 2015). This method was sensitive in detecting the morphological topology (e.g., He et al., 2009; Zhang et al., 2011). A wide range of the sparsity (S) thresholds was set up with a wide range for each correlation matrix. The range values of S were set to make sure that the thresholded networks were measured for the small-worldness with sparse properties and had the minimum number of spurious edges (Watts and Strogatz, 1998). Consequently, threshold values, ranging between 0.10 and 0.34 with an interval of 0.01, were used. All global and nodal network metrics were measured at each sparsity value. For each network metric, the area under the curve (AUC) was calculated to reflect measures across the sparsity parameter S. Nodes with statistically significant differences in network efficiency of local brain regions between the two groups of subjects were visualized using the BrainNet Viewer software (Version 1.6^3^).

At the global level, global efficiency (E_*glob*_), characteristic path length (Lp), normalized characteristic path length (λ), clustering coefficient (Cp), normalized clustering coefficient (γ), and small-worldness (σ) were calculated. At the nodal level, the nodal degree, nodal efficiency (E_*loc*_), and nodal betweenness were computed (Rubinov and Sporns, 2010). Briefly, Cp reflects the local interconnectivity extent. Lp measures the average distance or routing efficiency between any pair of network nodes, with higher values indicating lower routing efficiency. Small-world attributes (γ, λ, and σ) indicate the degree of organization of the small world. E_*glob*_ indicates the global efficiency of parallel information transfer in the network. E_*loc*_ reflects the communication efficiency among the first neighbors of a node. The nodal degree reflects the capacity to communicate information. The nodal efficiency indicates the nodal efficiency of parallel information transfer. The nodal betweenness centrality means the influence of a node over information flow between other nodes in the network. Detailed information on these metrics was previously provided (Rubinov and Sporns, 2010).

## Statistical Analysis

Group comparisons of demographic and clinical data were conducted using IBM SPSS software (IBM SPSS Statistics for Windows, Version 24.0, IBM Corp., Armonk, NY, United States). Two-tailed independent-sample *t*-tests and chi-square tests were performed. The threshold was set at *P* < 0.05.

A non-parametric substitution test was used for morphological topology metric comparison analysis (e.g., Zhang et al., 2011; Lei et al., 2015). The AUC of each network metric was calculated across the S values to determine differences. All values were randomly reclassified into two groups, and the mean difference between the two groups was calculated for each network metric for multiple comparisons. This randomization procedure was repeated 10,000 times. The 95th percentile of each distribution was used as the threshold for significance testing. In addition, the Benjamini–Hochberg procedure was used to test for nodal centrality and to correct for multiple comparisons by controlling for false discovery rate (FDR) (Benjamini et al., 2001).

As children’s brains are undergoing development, we proposed to analyze the age and diagnosis interaction effect. This analysis used a general linear model to examine the interaction effects of the network with age and diagnosis at global and node levels. In this analysis, gender and years of education were used as covariates, diagnosis, and age and the interaction of diagnosis and age were the dependent variables. The results were corrected for FDR, setting *P* < 0.05, as the threshold. We also analyzed the correlation between the morphological metrics and clinical data. For the global and nodal attributes, where differences were detected earlier, correlation analysis was performed with the clinical scale separately. The above analysis was conducted by the IBM SPSS software (version 24.0).

## Results

### Clinical Information

The basic clinical information and YGTSS scores are shown in Table 1. Age and sex were matched between the TS and HCs (*p* > 0.05).

**TABLE 1:** Demographic characteristics of patients with Tourette’s syndrome (TS) and healthy controls (HCs).

Characteristics	Tourette’s syndrome		Healthy control		
	Mean	SD	Mean	SD	*P*-value
Age (years)	7.97	1.91	8.67	2.63	0.136
Education (years)	2.24	1.70	2.89	1.72	0.642
Gender (Male/Female)	36/23	NA	48/39	NA	0.483
Age at onset (years)	5.38	2.79	NA	NA	NA
Disease duration (month)	23.00	21.42	NA	NA	NA
Total YGTSS score	22.84	7.56	NA	NA	NA
Total motor tic score	13.96	4.32	NA	NA	NA
Total phonic tic score	8.88	4.60	NA	NA	NA
					

### Brain Network Properties Alterations

Compared to HCs, patients with TS indicated a significant increase in E_*glob*_ (*p* = 0.012) and a decrease in λ (*p* = 0.027) and Lp (*p* = 0.025). Increased E_*global*_ means increased global efficiency of parallel information transfer in the network, lower Lp values indicate higher routing efficiency. No significant differences were identified in the Eloc (*p* = 0.063), Cp (*p* = 0.217), γ (*p* = 0.267), or σ (*p* = 0.435). The *p*-value has been FDR corrected.

### Differences in Nodal Brain Network Metrics

**FIGURE 1:**
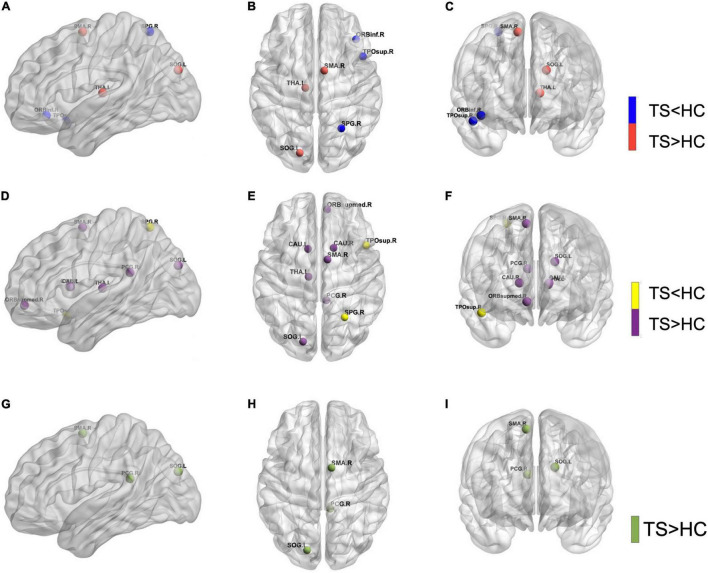
The significant alternations of nodal betweenness **(A–C)**, nodal degree **(D–F)**, and nodal efficiency **(G–I)** of gray matter (GM) in pediatric Tourette syndrome and healthy controls. CAU, caudate nucleus; HC, healthy control; L, left; ORBinf, inferior frontal gyrus, orbital part; ORBsupmed, superior frontal gyrus, medial orbital; PCG, posterior cingulate gyrus; R, right; SMA, supplementary motor area; SOG, superior occipital gyrus; SPG, superior parietal gyrus; THA, thalamus; TPOsup, temporal pole: superior temporal gyrus; TS, Tourette syndrome.

The nodal betweenness, nodal degree, and nodal efficiency differences are presented in Table 2. The significantly different regions included the right frontal lobe, right temporal lobe, right parietal lobe, right post-cingulum, bilateral caudate nucleus, and left thalamus (Figure 1).

**TABLE 2:** Regions showing altered node centrality (including nodal betweenness, nodal degree, and nodal efficiency) in patients with TC and HCs.

Brain regions	*P*-values		
	Nodal betweenness	Nodal degree	Nodal efficiency
**TS > HC**			
SMA.R	0.0371*	0.0006*	0.0006*
ORBsupmed.R	0.0645	0.0027*	0.0080
PCG.R	0.3837	0.0002*	0.0002*
SOG.L	0.0022*	0.0002*	0.0004*
CAU.L	0.0073	0.0024*	0.0025
CAU.R	0.1313	0.0024*	0.0043
THA.L	0.0002*	0.0004*	0.1222
**TS < HC**			
ORBinf.R	0.0022*	0.0159	0.1013
SPG.R	0.0002*	0.0041*	0.0621
TROsup.R	0.0031*	0.0020*	0.0039

*Significant differences with FDR correction were applied to each nodal measure. A permutation test was conducted to get the p-values. CAU, caudate nucleus; HC, healthy control; L, left; ORBinf, inferior frontal gyrus, orbital part; ORBsupmed, superior frontal gyrus, medial orbital; PCG, posterior cingulate gyrus; R, right; SMA, supplementary motor area; SOG, superior occipital gyrus; SPG, superior parietal gyrus; THA, thalamus; TPOsup, temporal pole: superior temporal gyrus; TS, Tourette’s syndrome.

### Relationships Between Topological Metrics and Clinical Data

**FIGURE 2:**
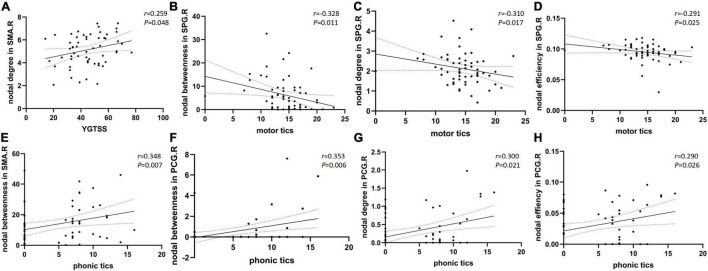
Scatter plots with 95% confidence interval (CI) of nodal brain network properties of different regions compared to YGTSS total scores **(A)** and sub scores of motor tics **(B–D)** and phonic tics **(E–H)**.

**FIGURE 3:**
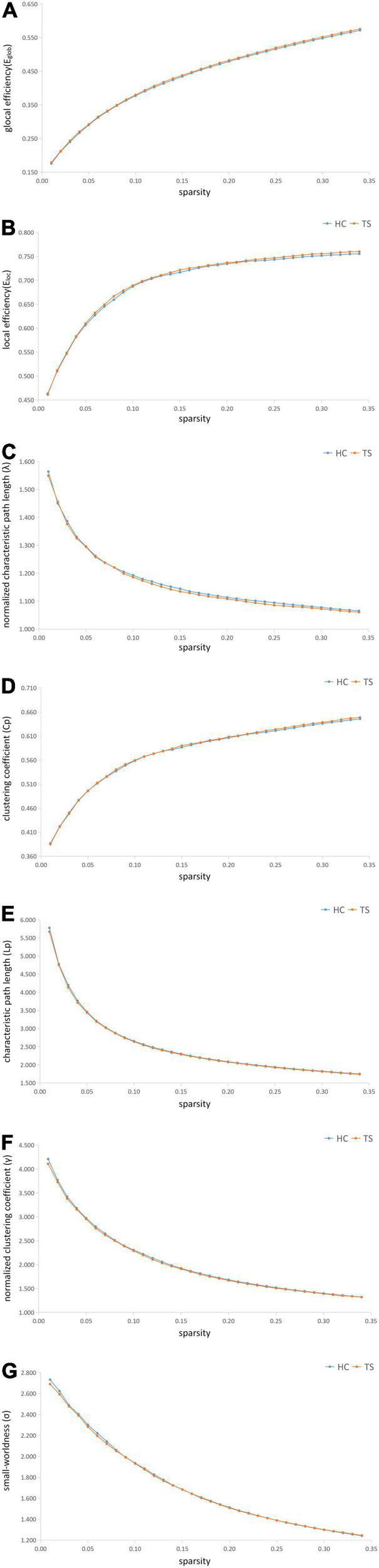
The metrics of the structural connectome of the sparsity threshold in global efficiency **(A)**, local efficiency **(B)**, normalized characteristic path length (λ) **(C)**, clustering coefficient (Cp) **(D)**, characteristic path length (Lp) **(E)**, normalized clustering coefficient (γ) **(F)**, small-worldness (σ) **(G)**, respectively.

Nodal betweenness, nodal degree, and nodal efficiency in the right superior parietal gyrus were negatively correlated with the motor tics scores of the YGTSS (*r* = −0.328, *p* = 0.011; *r* = −0.310, *p* = 0.017; *r* = −0.291, and *p* = 0.025, respectively). Nodal betweenness, nodal degree, and nodal efficiency in the right posterior cingulate gyrus were positively correlated with the YGTSS phonic tics scores (*r* = 0.353, *p* = 0.006; *r* = 0.300, *p* = 0.021; *r* = 0.290, and *p* = 0.026, respectively). Nodal betweenness in the right supplementary motor area was positively correlated with the YGTSS phonic tics scores (*r* = 0.348, *p* = 0.007). The nodal degree in the right supplementary motor area was positively correlated with the YGTSS phonic tics scores (*r* = 0.259, *p* = 0.048). The correlations were exhibited in Figure 2. The metrics of the structural connectome of the sparsity threshold were shown in Figure 3.

### Age and Diagnosis Interaction on Network Properties

The E_*glob*_, λ, Lp revealed no significant main effect of age and diagnosis interaction at the global level. At the nodal level, our findings revealed no significant main effect of diagnosis-by-age interaction on nodal betweenness, nodal degree, or nodal efficiency (Table 3).

**TABLE 3:** Linear modeling of age effects on node-level average controllability in drug-naïve patients with TS and HCs.

Source	Dependent variable	Type III sum source of squares	df	Mean square	F value	*P*-value
Diagnosis*age	E_*glob*_	1.25E-06	1	1.254E-6	0.363	0.548
	E_*loc*_	9.67E-07	1	9.674E-7	0.119	0.730
	Cp	3.53E-06	1	3.529E-6	0.353	0.553
	γ	3.92E-05	1	3.922E-5	0.027	0.869
	λ	7.26E-05	1	7.260E-5	3.294	0.072
	Lp	3.34E-05	1	3.342E-5	0.301	0.584
	Nodal betweenness in ORBinf.R	1.020	1	1.020	0.018	0.895
	Nodal betweenness in SMA.R	269.783	1	269.783	1.779	0.184
	Nodal betweenness in SOG.L	11.679	1	11.679	0.039	0.844
	Nodal betweenness in SPG.R	8.774	1	8.774	0.094	0.759
	Nodal betweenness in TPOsup.R	93.188	1	93.188	0.487	0.486
	Nodal degree in SMA.R	1.221	1	1.221	0.702	0.404
	Nodal degree in ORBsupmed.R	4.325	1	4.325	0.753	0.387
	Nodal degree in PCG.R	0.050	1	0.050	0.505	0.479
	Nodal degree in SOG.L	0.015	1	0.015	0.005	0.946
	Nodal degree in SPG.R	0.042	1	0.042	0.044	0.834
	Nodal degree in CAU.L	0.678	1	0.678	0.800	0.373
	Nodal degree in CAU.R	1.656	1	1.656	1.610	0.207
	Nodal degree in TPOsup.R	2.082	1	2.082	0.891	0.347
	Nodal efficiency in SMA.R	5.836E-5	1	5.836E-5	0.401	0.528
	Nodal efficiency in PCG.R	0.000	1	0.000	0.436	0.510
	Nodal efficiency in SOG.L	1.985E-6	1	1.985E-6	0.008	0.931

CAU, caudate nucleus; HC, healthy control; L, left; ORBinf, inferior frontal gyrus, orbital part; ORBsupmed, superior frontal gyrus, medial orbital; PCG, posterior cingulate gyrus; R, right; SMA, supplementary motor area; SOG, superior occipital gyrus; SPG, superior parietal gyrus; THA, thalamus; TPOsup, temporal pole: superior temporal gyrus; TS, Tourette’s syndrome. *Means the interaction effect of age and diagnosis.

## Discussion

This study explored gray matter topological alterations in drug-naïve pediatric patients with TS. Patients with TS showed a significant increase in E_*global*_ and a decrease in λ and Lp. Altered nodal characteristics were observed, some of which were correlated with the YGTSS total scores, phonic tics scores, and motor tics scores. Nodal characteristic metrics are mainly involved in the cortex-striatum-thalamus-cortex (CSTC) circuit. This result is consistent with the results of previous structural studies. Furthermore, this result explores the structural abnormalities from a topological perspective.

The function of the supplementary motor area (SMA) is to control movement (Chen et al., 2010). The SMA can produce complex movement synergies and vocalizations. In the present study, the YGTSS total score was positively correlated with the nodal degree in the right SMA. In addition, phonic tics scores were positively correlated with nodal betweenness in the right SMA. The total tic severity increased as the nodal degree increased, and the phonic tic severity increased as the nodal betweenness increased. The nodal degree indicates the ability of information communication, and the nodal betweenness reflects the interaction between a node and the surrounding nodes. SMA plays a role in maintaining postural stability and physical coordination (Fujimoto et al., 2014). The SMA is closely linked to the basal ganglia. The SMA projects to both the presenter’s motor cortex and spinal cord (Tanji, 1994). Therefore, the SMA may be associated with motor tic and compulsive behaviors in TS (Tübing et al., 2018). Studies with probabilistic fiber tractography revealed reduced connectivity between the SMA and basal ganglia, as well as between frontal cortico-cortical circuits (Cheng et al., 2013) in patients with TS.

In this study, we found that nodal betweenness and nodal degree increased in the left thalamus of patients with TS. Previous structural studies showed an increased volume of the thalamus in both adults and children with TS (Miller et al., 2010). In patients with TS, the altered volume of the thalamus may be related to compensatory mechanisms related to its control of twitching and the formation of overactive motor circuits (Greene et al., 2017). The thalamus is the core nucleus that connects the peripheral tissues, is involved in the interactions between the cortical connections, and accelerates the synchronous oscillatory activity of the functional areas of the cortex (Benarroch, 2015). Convulsions may be related to the CSTC circuit (Draper et al., 2016). Studies have suggested that thalamic-striatal projections may act as positive reinforcement for striatal neurons, which have behaviorally selected roles. They monitor “top-down” control by modulating the activity of cortico-basal ganglia loops (Kimura et al., 2004; Smith et al., 2004).

We observed that nodal degree and nodal efficiency increased in the right posterior cingulate gyrus in patients with TS. The posterior cingulate gyrus is metabolically active and has extensive connections with surrounding brain regions (Leech and Sharp, 2014). It is temporally and spatially associated with common activities. The cingulate gyrus is a component of the limbic lobe and is associated with emotion, learning, and memory. The posterior cingulate gyrus amplifies tic impulses more than inhibits them. Structural MRI showed cortical thinning and/or below-normal volume in the cingulate cortex, correlating with tic severity. Moreover, in the posterior middle cingulate cortex, dorsal posterior cingulate cortex, and ventral posterior cingulate cortex, cortical thickness is a candidate biomarker shared across siblings with TS (O’Neill et al., 2019). This study indicated the right posterior cingulate gyrus played an essential role in topological perspective.

In this study, the nodal degree increased in the bilateral caudate nucleus in the TS group compared with that in the healthy controls. The caudate nucleus is part of the striatum, and its role includes correlating motor behavior with spatial information, as well as limb posture maintenance, speed, and accuracy of directed movements (Takakusaki et al., 2004; Turner and Desmurget, 2010). A previous study showed caudate nucleus volume reduction in both adults and children with TS (Gerard and Peterson, 2003). Our study showed a nodal degree alteration may result in tics.

The nodal betweenness and nodal degree in the superior parietal lobule were lower in patients with TS than in those of healthy controls. Motor tics scores were positively correlated with nodal betweenness, degree, and efficiency in the superior parietal lobule. The superior parietal lobule is involved in spatial orientation, which may be related to the tics. Generally, the nodal brain network properties were in the circuit.

While previous studies have explored brain structural abnormalities in TS from a structural perspective, our study explored structural topological alterations in TS. Brain structural abnormalities underlie the altered brain topology. Our study is consistent with previous structural studies that have identified altered brain structure topology in the CSTC circuit.

As the brain develops in children, diagnosis and age interaction analyses were conducted. The results showed no significant main effect of diagnosis-by-age interactions on the global or nodal brain network properties. Therefore, the changes in the global or nodal brain network properties between the patients with TS and healthy controls were not related to age.

## Conclusion

Using a novel gray matter morphological topology method and a moderate patient group, we showed alterations in global or nodal brain topological properties in patients with TS compared with those of healthy controls. The nodal brain network properties are included in the CSTC circuit. These alterations were not affected by age of the developing brains of children.

## Data Availability Statement

The raw data supporting the conclusions of this article will be made available by the authors, without undue reservation.

## Ethics Statement

The studies involving human participants were reviewed and approved by the Human Ethics Committee of West China Second University Hospital of Sichuan University. Written informed consent to participate in this study was provided by the participants’ legal guardian/next of kin.

## Author Contributions

YL, XC, and HQ proposed the conception and design of the study and wrote the manuscript. YL and XLL analyzed the data. YJ, FJ, XH, and GN analyzed the clinical data and imaging data. XSL and CF collected the imaging data and analyzed the data. HZ and XC assessed the patients and collected the clinical data. All authors contributed to the article, critically reviewed the manuscript, and approved the submitted version.

## Conflict of Interest

The authors declare that the research was conducted in the absence of any commercial or financial relationships that could be construed as a potential conflict of interest.

## Publisher’s Note

All claims expressed in this article are solely those of the authors and do not necessarily represent those of their affiliated organizations, or those of the publisher, the editors and the reviewers. Any product that may be evaluated in this article, or claim that may be made by its manufacturer, is not guaranteed or endorsed by the publisher.

## Footnotes

1
http://www.fil.ion.ucl.ac.uk/spm/software/spm12/

2
http://www.mathworks.com/matlabcentral/fileexchange/14034-kernel-density-estimator

3
https://www.nitrc.org/projects/bnv/
